# Nutrition Support Adequacy in Children with Biliary Atresia After Liver Transplant

**DOI:** 10.3390/nu18010133

**Published:** 2025-12-31

**Authors:** Nicole Knebusch, Manpreet Virk, Moreshwar S. Desai, Marwa Mansour, Stacey Beer, Brittany Pearo, Kelby Fuller, Krupa Mysore, John Goss, Thomas Fogarty, Fernando Stein, Jorge A. Coss-Bu

**Affiliations:** 1 Division of Critical Care Medicine, Department of Pediatrics, Baylor College of Medicine, Houston, TX 77030, USA; nknebusch@ufm.edu (N.K.);; 2 Texas Children’s Hospital, Houston, TX 77030, USA; 3 Imperial College of London, London SW7 2AZ, UK; 4 Division of Pediatric Gastroenterology, Hepatology and Nutrition, Department of Pediatrics, Baylor College of Medicine, Houston, TX 77030, USA; 5 Department of Clinical Nutrition Services, Texas Children’s Hospital, Houston, TX 77030, USA; 6 Department of Surgery, Baylor College of Medicine, Houston, TX 77030, USA

**Keywords:** pediatrics, nutrition support, intensive care, liver transplant, biliary atresia

## Abstract

**Background:** The nutrition support of children with biliary atresia after liver transplant is affected by multiple factors, and a connection between these factors and conditions present before transplant can potentially make the nutrition support more challenging. We aim to assess the adequacy of nutrition support, specifically energy and protein, during the first week of admission to the Pediatric Intensive Care Unit (PICU) in children after liver transplant secondary to biliary atresia. **Methods**: We performed a retrospective cohort study of 138 patients [13.9 median (9–33.4) IQR months; 62% female] with a diagnosis of biliary atresia admitted to the PICU after liver transplantation at Texas Children’s Hospital over a 14-year study period. We obtained nutrition adequacy of enteral and parenteral nutrition support for the first week after transplant during their PICU admission. **Results**: Goal adequacy was reached at the end of the first week of admission when combined enteral and parenteral nutrition support was provided (median 98% for energy and 101% for protein). Infants achieved significantly higher adequacies than older children during the first week (136% < 1 year vs. 0% > 1 year, *p* < 0.001 for calories, and 157% < 1 year vs. 0% > 1 year for protein; *p* < 0.01). **Conclusions**: These findings highlight the complex nutritional challenges faced by this population, and strategies are needed to meet the unique needs of children after liver transplantation.

## 1. Introduction

Biliary atresia (BA) is the most common cause of neonatal cholestasis. It is a progressive obstructive disease of the intrahepatic and extrahepatic biliary tree, accompanied by structural changes and fibrosis [1,2,3]. Biliary obstruction leads to severe cholestasis, rapid progression of fibrosis and subsequent cirrhosis and the need for a liver transplant [4]. It is a rare disease of early infancy with an unknown etiology, and an incidence of around 8000–18,000 live births worldwide [1,5]. Specific mechanisms have been proposed for the etiology of BA, including perinatal viral infection, toxin exposure, altered immune response, or abnormal bile duct development [1].

Some of the common indications for liver transplant include inborn errors of metabolism, autoimmune diseases, and Wilson’s disease, with BA being the disease of interest in this research as the leading indication for liver transplant in children [5].

One of the indications for liver transplantation in children with BA is failure to thrive and moderate to severe malnutrition, which is common in these children [1]. Several factors contribute to malnutrition and poor linear growth in children with BA, including inadequate intake because of anorexia secondary to the inflammatory state and altered taste secondary to micronutrient deficiency, nausea, early satiety, fat malabsorption caused by decreased bile flow and altered recirculation of biliary salts, impaired micelle absorption and formation, altered carbohydrate utilization and stores, impaired gluconeogenesis and hypermetabolism with pro-inflammatory cytokines as drivers and many other things [2,6]. Children with BA and end-stage liver disease are also at risk of developing sarcopenia and metabolic bone disease due to abnormal metabolism, which can adversely affect their growth and functionality in the long term [7]. They are also commonly affected by stunting or decreased linear growth, malnutrition, and growth hormone resistance [8]. In children, nutritional status is commonly assessed using anthropometric measures, including weight-for-age, height-for-age, and weight-for-length or body mass index z-scores, which help characterize acute and chronic malnutrition as well as growth impairment [1,2,9].

After liver transplantation, children are admitted to the pediatric intensive care unit (PICU) for initial post-transplant treatment. Nutrition support will vary according to baseline nutritional status, accompanying diseases and comorbidities, hemodynamic stability, and respiratory support [9]. Children admitted to the PICU require nutrition support and monitoring due to the risk of losing lean body mass secondary to protein catabolism and dysregulation of energy metabolism [6]. So, children post-liver transplantation do not only carry the burden of previously altered metabolism, but they also bear other burdens due to neuroendocrine and metabolic responses to stress [10]. Given that pediatric liver transplant recipients frequently exhibit preexisting malnutrition, timely initiation and vigilant monitoring of nutritional support are essential [11]. Immediately following surgery, energy demands increase, and meeting the necessary calories and protein intake is imperative. Adequate nutritional support can help decrease the risk of infection, support the healing process, reduce body mass loss, and reduce the risk of muscle atrophy and other complications that can alter recovery after a liver transplant [12].

One way to objectively assess energy and protein provision after surgery is to evaluate nutritional adequacy during the first week of PICU admission. In this context, nutritional adequacy is defined as the proportion of prescribed energy and protein requirements that are delivered to the patient, typically expressed as a percentage of estimated needs over a defined time period [9,13]. Nutrition adequacy studies in PICU patients have demonstrated that achieving at least 60% of energy and protein requirements during the first week of PICU admission is associated with decreased mortality [9]. A multicenter analysis of 1844 critically ill pediatric patients by Bechard et al. showed that patients who reached the goal of 60% adequacy in calories and protein during the initial week of admission had a lower mortality risk [9]. Other similar studies have been performed to assess nutritional adequacy in PICU patients, including a study by Mehta, N.M. et al. of 1245 children, which found that achieving at least 66% of protein adequacy when provided via the enteral route was associated with decreased 60-day mortality [13]. Nutritional adequacy during the first week of admission to the PICU in liver transplant recipients aims to mitigate the effects of surgical stress and counteract the effects of high-dose immunosuppressants [12]. It is crucial to further study how the first week of nutrition support affects long-term outcomes, such as growth and development, given that this is a nutritionally vulnerable population and that nutritional status and support significantly impact patient and transplant outcomes.

We aim to assess the adequacy of nutrition support, specifically energy and protein, during the first week of admission to the PICU in children after liver transplant secondary to BA. We hypothesize that achieving ≥ 60% goal adequacy for calories and protein within 7 days of admission is feasible only when combined enteral and parenteral nutrition support is used in this patient population.

## 2. Materials and Methods

### 2.1. Study Design

This study was a single-center retrospective observational cohort study of children, <18 years of age, with BA admitted to the Pediatric Intensive Care Unit (PICU) after liver transplantation at Texas Children’s Hospital in Houston, TX, USA, from 2011 to 2024. Inclusion criteria consisted of all pediatric patients (<18 years of age) with a diagnosis of biliary atresia who were admitted to the PICU following liver transplantation during the study period. Patients were excluded if they underwent liver transplantation for indications other than biliary atresia. The diagnosis of biliary atresia was established prior to transplantation and documented in the institutional transplant database. Patient demographic and clinical data were collected from Texas Children’s Hospital’s database and electronic medical records (EMR). Extracted data included information from the admission and follow-up for up to one year after transplant. Demographic data included age, sex, and race. Clinical data included anthropometric measurements (pre-transplant weight, height, or length), primary diagnosis, and surgical data (surgery duration, anesthesia duration, transplantation technique, mechanical ventilation (MV) hours, and length of stay (LOS). The Baylor College of Medicine Institutional Review Board approved the study H-39403 on 17 March 2021.

### 2.2. Nutritional Assessment

Nutritional status was analyzed using anthropometric measurements and the Centers for Disease Control and Prevention (CDC) growth charts for children older than 2 years of age, and the World Health Organization (WHO) growth charts for children less than 2 years of age [14,15]. We included height/length-for-age (HFA) Z-score, weight-for-age (WFA) z-score, and weight-for-length/Body Mass Index-for-age (BMI/A) z-scores as age-appropriate [14,15]. For this study, we will refer to BMI/A as WFL. The nutritional status of the patients was assessed at the time of liver transplant. Underweight was defined as WFA z-score ≤ −2.0, acute malnutrition as WFL z-score ≤ −2.0, and stunting or chronic malnutrition as HFA z-score ≤ −2.0.

### 2.3. Nutritional Support

Nutrition support was calculated for all children, and nutrition adequacy was based on the guidelines of the American Society of Parenteral and Enteral Nutrition (ASPEN) and the Society of Critical Care Medicine (SCCM) for critically ill children [9]. Following SCCM and ASPEN guidelines, the basal metabolic rate was calculated using Schofield’s equation to estimate energy needs, and the protein goal was established as 1.5 g per kilogram per day (g/kg/day) [9]. Nutrition support intake was calculated for all children, including enteral and parenteral nutrition. We did not quantify oral feeding intake. Nutrition adequacy was calculated as [(nutritional intake/estimated needs) × 100].

### 2.4. Statistical Analysis

Quantitative analyses of descriptive data are presented as means and standard deviations (SD) or medians with interquartile ranges (IQR, 25th to 75th). Categorical variables are reported as counts and percentages. Mann–Whitney, Chi-square, and Fisher’s exact tests were used as appropriate for the comparison of continuous and categorical variables. Statistical significance was established a priori as *p* < 0.05. Statistical analysis was performed with Stat View Version 5.0.1 (SAS Institute Inc., Cary, NC, USA).

## 3. Results

A total of 368 patients were admitted for liver transplants at Texas Children’s Hospital from 2011 to 2024. Out of those, 138 patients were diagnosed with BA. The median age was 13.9 (9.0–33.4) months, weight was 10.0 (8.4–14.0) kilograms, length or height was 75 (67.0–92.3) centimeters, and 62% (*n* = 86) of patients were female. The racial distribution was: 13.8% of patients identified as African American, 2.9% as Asian, 58.0% as White, and 5.8% identified as other. 19.6% were of Hispanic ethnicity.

The median and 25th–75th IQR for Pediatric End-Stage Liver Disease (PELD) was 26.0 (15.0–30.3), total surgery time including anesthesia time 355 (336–393) mins, 1.0 (0.5–20) mechanical ventilation (MV) days, pediatric intensive care unit (PICU) length of stay (LOS) of 7.0 (3.0–25.5) days and hospital LOS of 24 (9.0–63) days.

Regarding the nutritional status of patients, the prevalence of stunting (HFA < −2 z-scores) was 21.7%, and the prevalence of underweight (WFA < −2 z-scores) was 10.2%. When analyzing anthropometric data with WFL or BMI/A z-scores, 87.7% of patients had a normal nutritional status, 1.4% were acutely malnourished, and 10.9% were obese. The median basal metabolic rate was 49.4 (45.5–53.5) kcal/kg/day, calculated using Schofield’s equation. See Table 1.

We compared clinical variables based on nutritional status at the time of admission and found that patients admitted to the PICU with stunting (HFA < −2 z-score) required a total of 6.2 (0.5–31.7) days of mechanical ventilation, compared to 0.6 (0.5–11) days for those patients without stunting (*p* = 0.0117). They also experienced a longer PICU length of stay of 15.2 (3.6–51) days versus 6.0 (3.0–21.7) days when compared to children without stunting (*p* = 0.0211); however, no significant difference was found between stunting and overall hospital stay (*p* = 0.068). No other significant associations were identified with different types of malnutrition.

### 3.1. Overall Nutrition Adequacy

The nutrition support and intake were calculated for days 1, 3, 5, and 7 after liver transplant. All calculable nutrition support was evaluated, including enteral nutrition support and parenteral nutrition support. Per Os (PO), nutrition was not included in the calculation. Overall nutritional intake increased progressively over the first week following liver transplantation, with total caloric and protein adequacy approaching recommended targets when all nutrition sources were considered. On day 1, children received a median IQR of 0.00 (0.00–14.2) kilocalories per kilogram per day (kcal/kg/day), and for protein, 0.00 (0.00–0.40) g/kg/day. On day 3, this number increased to 32.0 (0.00–60.6) kcal/kg/day and 1.00 (0.00–2.40) g/kg/day of protein. By day 5, children received 44.9 (0.00–73.4) kcal/kg/day and 1.45 (0.00–2.60) g/kg/day of protein. By the end of the week, children received 49.8 (0.00–75.0) kcal/kg/day and 1.50 (0.00–2.60) g/kg/day of protein; these median values are equivalent to the basal metabolic rate calculated by Scholefield’s equation and the recommended amount of protein by SCCM and ASPEN guidelines [9]. See Table 2.

Nutrition support adequacy median values on the day of admission were 0% for calories and 0% for protein. By day 3 of PICU admission, the goal of adequacy for calories and protein was reached when total nutrition support was considered. For total calories, adequacy was 60%, and for total protein, adequacy was 69%. On day 5, 93% of caloric adequacy was reached, and 97% of protein adequacy. By day 7, 98% of caloric adequacy was achieved and 101% of protein adequacy. For the analysis of enteral nutrition support for caloric and protein goals throughout the initial week of admission, the median values for adequacy of enteral nutrition for calories for days 1, 3, 5, and 7 were 0%, 0%, 4%, and 6%, respectively. Enteral protein intake median values for adequacy on days 1, 3, 5, and 7 were 0%, 0%, 4%, and 5%, respectively, and remained below 10% throughout the week. See Table 3.

In addition to median adequacy values, we evaluated the proportion of patients who achieved the 60% caloric and protein intake thresholds during the first week after transplantation. The percentage of patients who reached at least 60% of the total calories goal during the first seven days of admission on days 1, 3, 5, and 7 was 17.5%, 49.6%, 62%, and 61%, respectively. When analyzing enteral nutrition support for calories, 0% reached the 60% goal by day 1, increasing slightly to 3.6% by day 3. On day 5, 18% of patients achieved goal adequacy for calories, and by day 7, 23% reached goal adequacy when enteral nutrition support alone was considered.

For protein, the percentage of patients who reached 1.5 g/kg/day of total protein intake during the first week of admission was 12.3% on day 1, 43.5% on day 3, 50% on day 5, and 53% on day 7. For total protein adequacy, the percentages of patients who reached at least 60% adequacy on days 1, 3, 5, and 7 were 20%, 51%, 62%, and 62%, respectively. When considering only enteral nutrition support, 60% of protein goals were reached by 0% of patients on day 1, 3.4% on day 3, 18% on day 5, and 23% on day 7.

### 3.2. Age-Related Differences

To assess whether nutritional delivery and clinical outcomes differed by age, we compared patients younger than one year with those older than one year at the time of transplant. See Table 4.

We compared clinical variables between patients younger than 1 year at transplant (*n* = 58, 42%) and those older than 1 year at transplant (*n* = 80, 58%). Mechanical ventilation days were 13.0 (2.0–31.7) in children less than 1 year of age vs. 0.5 (0.5–2.0) days in children older than 1 year of age, *p* < 0.0001. Both PICU and hospital length of stay showed a statistically significant difference, with PICU LOS in patients younger than 1 year of age being 20.3 (9.0–48.2) days vs. 3.9 (2.0–7.8) days in older children, *p* < 0.0001; and hospital LOS in patients younger than 1 year of age was 62.0 (26.0–133.0) days vs. 12.6 (7.3–28.0) days in older than 1 year of age *p* < 0.0001. Stunting was present in 13 (16.3%) young children vs. 17 (29.3%) of older children, *p* = 0.09; 6 (10.3%) of children less than 1 year of age were underweight, while 8 (10.0%) of children over 1 year, *p* > 0.99, and 2 (3.5%) of children less than 1 year of age were acutely malnourished vs. 0 (0%) of children over 1 year of age, *p* = 0.17.

**Figure 1:**
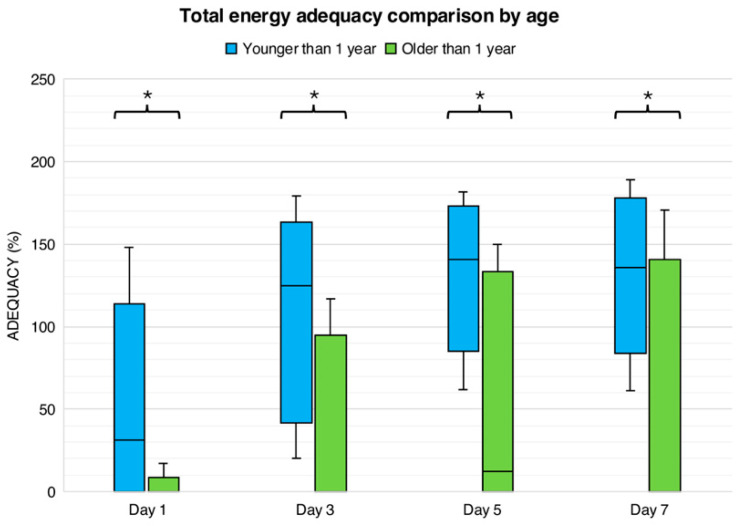
Total caloric adequacy comparison by age. Values represent the percentage of adequacy for calories based on age. Comparison by Mann–Whitney test. * *p* < 0.001.

We further evaluated age-based differences in caloric and protein adequacy over time, considering both total nutritional intake and enteral contributions. We compared goal adequacy achievement in children under 1 year (*n* = 58) and those over 1 year (*n* = 80). Figure 1 illustrates age-based differences in caloric adequacy over the first week of PICU admission. On day 1, enteral caloric adequacy was negligible in both age groups 0 (0–0)% whereas total caloric adequacy was significantly higher in children younger than one year compared with those older than one year (31 [0–79]% vs. 0 [0–0]%, *p* < 0.01).

By day 3, enteral calorie adequacy remained at 0 (0–0)% in both groups; however, a marked difference persisted in total caloric adequacy, with younger children achieving substantially higher adequacy than older children (125% vs. 0%, *p* < 0.01). Similar patterns were observed on days 5 and 7. On day 5, enteral adequacy increased modestly in children younger than one year (17 [0–51]%) but remained 0% in older children (*p* < 0.05), while total caloric adequacy continued to differ significantly between groups (141 [108–164]% vs. 12 [0–117]%, *p* < 0.01). On day 7, enteral caloric adequacy remained low overall but was higher among younger children (30 [0–66]% vs. 0 [0–65]%, *p* < 0.05). Total caloric adequacy at this time point was again significantly greater in children younger than one year (136 [107–167]%) compared with those older than one year (0 [0–110]%, *p* < 0.01).

**Figure 2:**
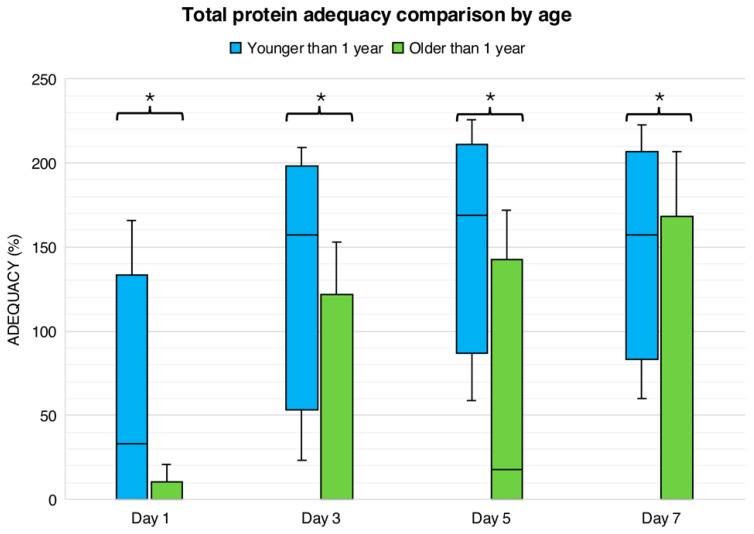
Total protein adequacy comparison by age. Values represent the percentage of adequacy for protein based on age. Comparison by Mann–Whitney test. * *p* < 0.001.

Figure 2 presents age-related differences in protein adequacy during the first week following liver transplantation. On day 1, enteral protein adequacy was 0 (0–0)% in both age groups. In contrast, total protein adequacy was significantly higher in children younger than one year, who achieved a median adequacy of 33 (0–101)%, compared with 0 (0–0)% in children older than one year (*p* < 0.0001). On day 3, enteral protein adequacy remained minimal in both groups (0 [0–11]% in younger children and 0 [0–6]% in older children). However, total protein adequacy continued to differ significantly by age, with younger children achieving markedly higher adequacy than older children (157% [84–187] vs. 0% [0–91], *p* < 0.01).

By day 5, enteral protein intake increased modestly in children younger than one year (12 [0–39]%) but did not differ significantly from older children (0 [0–48]%, *p* > 0.05). Total protein adequacy at this time point remained significantly greater in younger children (169 [115–196]%) compared with older children (18 [0–113]%, *p* < 0.01). On day 7, enteral protein adequacy remained low in both groups (21 [0–46]% in younger children vs. 0 [0–66]% in older children, *p* > 0.05), while total protein adequacy continued to be significantly higher among children younger than one year (157 [106–190]% vs. 0 [0–130]%, *p* < 0.01).

### 3.3. Enteral vs. Total Nutrition Contributors by Age

Finally, we examined age-specific differences in the proportion of patients achieving nutritional adequacy goals, distinguishing between total and enteral nutrition sources.

**Figure 3:**
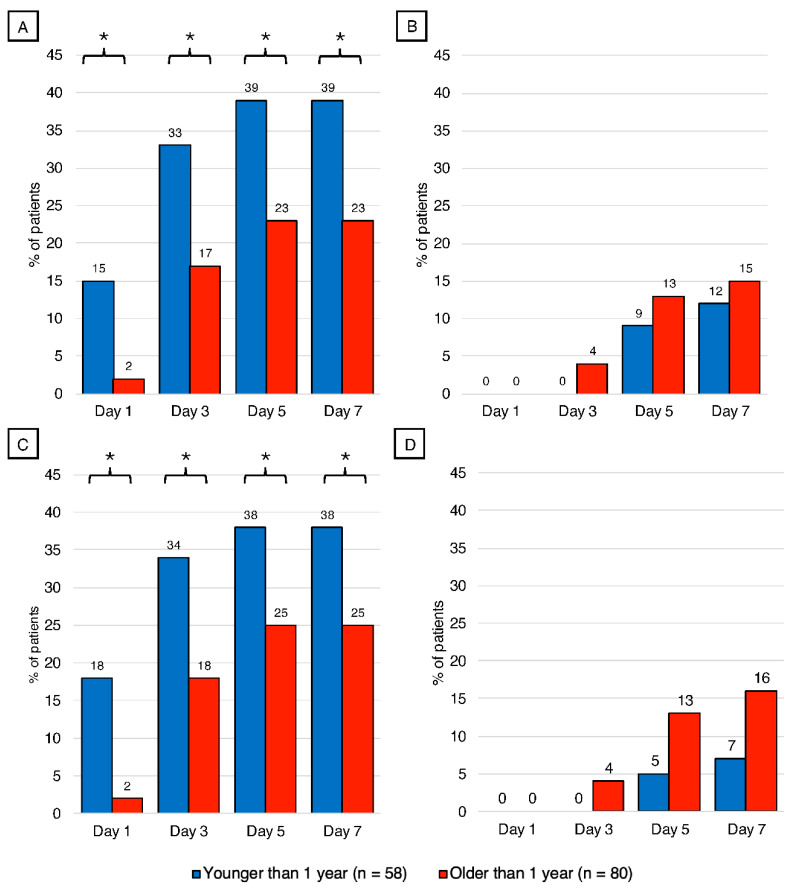
Percentage of patients that reached 60% goal adequacy of total and enteral caloric and protein adequacy based on age. Values represent the % of patients who reached 60% of the goal adequacy for both nutrients on days 1, 3, 5, and 7 of admission to the PICU for patients less than 1 year of age, compared with patients older than 1 year of age; analysis by Chi-Square. (**A**) Percentage of patients that reached total caloric goal adequacy, *p* < 0.001 for all days. (**B**) Percentage of patients reached enteral caloric goal adequacy, *p* > 0.05 for all days. (**C**) Percentage of patients that reached total protein goal adequacy, *p* < 0.001 for all days. (**D**) Percentage of patients that reached enteral protein goal adequacy, *p* > 0.05 for all days. * *p* < 0.001.

The percentages of children who reached goal adequacy (60%) during the first week of admission, compared by age, are illustrated in Figure 3. On day 1, 15% of children under 1 year reached goal adequacy for calories, compared to 2% of children older than 1 (*p* < 0.001). On day 3, 33% of children younger than 1 year reached caloric goal adequacy, compared to 17% of children older than 1 year (*p* < 0.001). On days 5 and 7, 39% of children under 1 year reached goal adequacy for calories, compared with 23% of older children (*p* < 0.001 for both days). On day 1, both groups reached 0% of goal adequacy for enteral nutrition support. For day 3, 4% of children older than 1 year reached caloric goal adequacy compared to 0% of children younger than one year (*p* > 0.05), and 9% of children younger than one year reached enteral calorie goal adequacy, compared to 13% of children older than 1 year on day 5 (*p* > 0.05). On day 7, 12% of children younger than one year reached enteral calorie goal adequacy, compared to 15% of children older than 1 year (*p* > 0.05). For total protein, 18% of children younger than 1 year reached the adequacy goal compared with 2% in children older than 1 year (*p* < 0.001). On day 3, 34% of children younger than 1 year reached 60% total protein adequacy, compared to 18% of children older than 1 year (*p* < 0.001). For days 5 and 7, 38% of children younger than 1 year reached total protein goal adequacy, compared to 25% of children older than 1 year (*p* < 0.001 for both days). For enteral protein, 0% of children younger than 1 year and 0% of those older than 1 year reached the protein adequacy goal. For days 3, 5, and 7, 0%, 5%, and 7% of children less than 1 year of age reached enteral protein goal adequacy, compared to 4%, 13%, and 16% of children older than 1 year of age (*p* > 0.05; for all days).

## 4. Discussion

Throughout the study period of approximately 13 years, we identified a total of 138 patients who were admitted to our unit after liver transplant due to BA. Considering that this is a rare disease that occurs in approximately 1 in 15,000 live births in the United States, the sample obtained is considered significant [1,3]. The nutritional support during the first week of PICU admission primarily relied on parenteral nutrition, with enteral nutrition contributing minimally to overall adequacy. By day 7, the median caloric and protein intakes had approached the recommended targets, with 98% and 101% adequacy, respectively. However, only 61% of patients achieved at least 60% of their caloric goals, and 53% met the protein intake target of 1.5 g/kg/day by day 7. Enteral nutrition alone accounted for less than 10% of total nutritional intake throughout the week, and only 23% of patients reached at least 60% of their caloric or protein goals via enteral nutrition by day 7.

These findings align with previous retrospective cohorts on nutrition support in critically ill children. Both studies reported that more than half of the children achieved nutritional adequacy for both calories and protein within the first week of admission to the PICU [16,17]. One study reported a comparison between enteral nutrition adequacy and total nutrition adequacy [17]. The study showed that, similar to our results, patients required parenteral nutrition to reach goal adequacy, while enteral nutrition was insufficient to achieve adequacy in these children. The study’s results, in conjunction with evidence from other studies, underscore the challenges of attaining early enteral nutrition goals in critically ill children. Miserachs et al. implemented a standardized feeding protocol for 49 children after liver transplantation. They managed to initiate enteral nutrition earlier with faster advancement of tube feeding (median value of 4 vs. 8 days) and a reduction in parenteral nutrition dependence from 75% to 47% without increasing adverse outcomes (*p* = 0.01) [18]. These results indicate a potential benefit of standardizing enteral nutrition use in the PICU to achieve its protective effects and that sufficient enteral protein is notably associated with reduced mortality [19,20]. A study of 467 adults evaluated whether early enteral nutrition was associated with a 1-year graft survival rate. The study found that 1-year graft survival in the early enteral nutrition group was 94.4%, compared with 85.4% in the non-early enteral nutrition group (*p* = 0.034) [21]. This study in adults highlights the importance of improving enteral nutrition support for children.

Another main finding of the study was the disparity in achieving goal adequacy between infants and older children. Throughout the first week of PICU, infants achieved a higher adequacy of both total calories and protein with the help of parenteral nutrition compared to children over one year (0% vs. 125% for total calorie adequacy and 0% vs. 157% for total protein in older vs. younger children, respectively). Among all patients, approximately 40% of those under 1 year reached ≥ 60% of their caloric or protein goals, compared with around 25% for older children, indicating that younger children received significantly better nutrition support than older children. These findings align with those reported by Mansour et al. in a cohort study on nutritional adequacy in children with neurological disorders [16]. This study found that younger children, those under 2 years old, had higher protein and calorie adequacy than older children admitted to the ICU. Another study compared the nutritional adequacy in children in the PICU and found an association between younger age and higher enteral nutrition support [20].

It is essential to consider that, although this finding suggests increased variability in nutrition support for older children in the PICU after liver transplant, other factors may confound these results. One factor was that younger children spent more time on mechanical ventilation (13.0 vs. 0.5) days; *p* < 0.0001, signifying that possibly older children were receiving oral feedings that were not quantified due to the nature of the study or that they were receiving another type of non-invasive respiratory support, such as positive airway pressure respiratory support, which in many centers is accompanied by nil-per-mouth [22]. Other potential factors may include a lack of standardization of nutrition support in older children and potential hesitancy in administering PN due to concerns about other complications related to the catheter [9,23,24]. Nonetheless, it is essential to address these issues in the future and rely on standard methods of providing nutrition support in children with BA after liver transplant.

In line with other studies, stunting and malnutrition have been linked to adverse outcomes in the PICU. We also observed an increased duration of mechanical ventilation and a longer PICU stay in children with stunting at the time of transplant [25,26,27]. A large retrospective cohort of 6377 pediatric patients by Nosaka et al. provided evidence that stunting, or chronic malnutrition, is associated with longer ICU stays (HR 0.85, 95% CI 0.81–0.90, *p* < 0.01) and increased mortality (3.6% vs. 1.4%, *p* < 0.01) [25]. Our cohort did not find any associations between PICU outcomes and other forms of malnutrition, such as those indicated by WFL/BMI or WFA. Nonetheless, other studies have consistently found this association [28,29,30].

In this cohort of pediatric liver transplant recipients with BA, we observed significant age-related differences in postoperative outcomes. Infants under 1 year experienced substantially longer mechanical ventilation duration (13.0 vs. 0.5 days). These findings are consistent with a multicenter cohort study evaluating pediatric liver transplants in the United States [31]. This study reported that younger age was a significant factor in the use of invasive mechanical ventilation, with use lasting more than 24 h (adjusted odds ratios [aORs] 0.90 [95% CI, 0.86–0.95] and 0.89 [95% CI, 0.85–0.95], respectively). Conversely, patients who did not require invasive MV postoperatively had a median age of 7 years [31]. As in other studies, younger children spent longer periods in the PICU than older children. In our study, children less than one year old had a PICU LOS of 20.3 days, compared to 3.9 days in older children (*p* < 0.0001). Others have shown similar results [32].

The calculated median basal metabolic rate of 49 kcal/kg/day in our patients is consistent with prior estimates in pediatric liver transplant candidates, supporting the use of formulas to estimate energy requirements to guide nutrition support [9]. Nonetheless, it is essential to note that energy requirements in this report were obtained with Schoefield’s equation, which includes weight as a variable. As previously mentioned, the weight is not an accurate measurement for these children [2]. In children with biliary atresia, who frequently have ascites, hepatomegaly, and edema, reliance on body weight may overestimate lean mass and lead to imprecision in calculated energy requirements. Incorporation of indirect calorimetry could strengthen future investigations, although its routine use in the PICU setting remains challenging [9]. One study of 11 patients with BA measured energy expenditure by indirect calorimetry and found that it was 29% higher than normal (68 kcal/kg/day) [33]. Nutritional and metabolic demands remain high in this population, especially among infants, which may contribute to prolonged ventilation and recovery periods.

In this study, one advantage of the retrospective design is the ability to include all patients with rare conditions who were available at the time of the study. Considering that this study was conducted at a center recognized as a national reference for liver transplantation in the United States, the data collected during this study period make it one of the largest of its kind in the country. Even so, the study has some limitations, including those inherent to its retrospective design. Accordingly, associations observed between nutritional status and clinical outcomes should be interpreted as descriptive, as this study was not designed to determine independent prognostic effects or causal relationships. In this study, we present the practices of a single center in the United States. Our center represents our institution’s nutritional support practices, and this study may not be generalizable to other centers. Another limitation of this study is that, given the length of the study period, there may have been changes in standard nutritional practices in the hospital and PICU. This is a natural phenomenon when it comes to policies and procedures, and may result in variations in results depending on the practice used. Additionally, the retrospective nature of the study means some data may be incomplete or missing, potentially affecting the accuracy of the results.

Despite these limitations, the study provides valuable insights into the practices and outcomes of liver transplantation at a leading center in the United States. Taken together, these findings highlight important implications for nutritional management following pediatric liver transplantation in children with biliary atresia. Although overall caloric and protein adequacy approached recommended targets by the end of the first week of PICU admission, this was largely achieved through parenteral nutrition, while enteral nutrition contributed minimally throughout the study period. In addition, older children were less likely to achieve nutritional adequacy compared with infants, suggesting potential variability in nutrition support practices across age groups. These results underscore the need for greater standardization of postoperative nutritional strategies, including earlier and more consistent advancement of enteral nutrition when clinically feasible. Such approaches may help reduce reliance on parenteral nutrition, address age-related disparities in nutritional management, and better support the metabolic demands of this population. Collectively, these findings may inform future institutional protocols and contribute to the development of evidence-based guidelines for nutritional management after pediatric liver transplantation.

## 5. Conclusions

Children with biliary atresia after liver transplantation achieved near-target energy and protein adequacy during the first week of PICU admission when combined enteral and parenteral nutrition support was provided. A significant finding of the study is that more than half of the patients with BA after liver transplant met approximately 100% of their energy and protein requirements within the first week of admission to the PICU, well above the goal adequacy recommendation. As previously stated, we hypothesized that this goal could be achieved through combined nutrition support, including enteral and parenteral nutrition, and this was confirmed by our results. Among those patients, only a minority achieved at least 60% of their calorie and protein goals using enteral nutrition alone. This study is consistent with previous studies on nutrition support during the PICU admissions of critically ill children, which also found that early enteral nutrition is challenging in the case of critically ill children.

Furthermore, we found a striking disparity in nutritional adequacy between infants and older children, with younger children achieving the highest levels of adequacy for both calories and protein, primarily due to the use of parenteral nutrition. There could be several confounding factors that account for the significant differences between these two groups, including variations in ventilatory support type, duration, and other variables, which may explain the substantial differences. Moreover, there were significant differences in postoperative outcomes by patient age; that is, younger children spent a longer time in the PICU and hospital after surgery. Several studies have demonstrated that recovery in young children is prolonged.

## Figures and Tables

**Table 1:** Patient characteristics.

Patient Characteristics	*n* = 138
Age (months)	13.9 (9–33.4)
Weight (kg)	10 (8.4–14)
Height (cm)	75 (67.0–92.3)
Female/Male	86/52
Ethnicity	
African American, *n* (%)	19 (13.8)
Asian, *n* (%)	4 (2.9)
Hispanic, *n* (%)	27 (19.6)
White, *n* (%)	80 (57.9)
Other, *n* (%)	8 (5.8)
Nutritional status	
Prevalence of stunting (HFA z-score < −2), *n* (%)	30 (21.7)
Prevalence of underweight (WFA z-score < −2), *n* (%)	14 (10.1)
Prevalence of acute malnutrition (WFL/BMI z-score < −2), *n* (%)	2 (1.4)
Prevalence of normal nutritional status (WFL/BMI z-score > −2–<+2), *n* (%)	121 (87.7)
Prevalence of obesity (WFL/BMI z-score > +2), *n* (%)	15 (10.9)
Basal Metabolic Rate by Schofield (kcal/kg/day)	49.4 (45.5–53.5)
Hospitalization period	
PELD at time of transplant	26 (15–30.3)
Surgery minutes	273 (244–303)
Anesthesia minutes	355.5 (336–393)
Mechanical ventilation (days)	1.0 (0.5–20)
PICU LOS (days)	7.0 (3–25.5)
Hospital LOS (days)	24 (9–63)
Mortality at 1 year, *n* (%)	6 (4.35)

Continuous variables are medians with interquartile ranges (25th–75th). Categorical variables are expressed as numbers and percentages. Kg: kilograms; cm: centimeters; HFA: height-for-age, WFA: weight-for-age, WFL: weight-for-length; BMI: Body mass index; PELD: Pediatric End-Stage Liver Disease; PICU: pediatric intensive care unit; LOS: length of stay.

**Table 2:** Total calorie and protein intake.

*n* = 138	Calorie Intake Kcal/kg/day	Protein Intake g/kg/day
Day 1	0.00 (0.00–14.2)	0.00 (0.00–0.40)
Day 3	32.0 (0.00–60.6)	1.00 (0.00–2.40)
Day 5	44.9 (0.00–73.4)	1.45 (0.00–2.60)
Day 7	49.8 (0.00–75.0)	1.50 (0.00–2.60)

Continuous variables are represented as medians with interquartile ranges (25th–75th). Kcal/kg/day: kilocalories per kilogram per day; g/kg/day: grams per kilogram per day.

**Table 3:** Caloric and protein adequacy after liver transplant.

*n* = 138	Caloric Adequacy (%)		Protein Adequacy (%)	
	Total	Enteral	Total	Enteral
Day 1	0 (0–33)	0 (0–0)	0 (0–31)	0 (0–0)
Day 3	60 (0–122)	0 (0–11)	69 (0–159)	0 (0–9)
Day 5	93 (0–148)	4 (0–51)	97 (0–173)	4 (0–42)
Day 7	98 (0–149)	6 (0–66)	101 (0–175)	5 (0–51)
BMR: 49.4 (45.5–53.5) kcal/kg/day and recommended protein intake: 1.5 g/kg/day				

Continuous variables are represented as medians with interquartile ranges (25th–75th). Adequacy was calculated as [(intake/prescription) × 100]. BMR: Basal metabolic rate by the Schofield equation.

**Table 4:** Comparison of clinical variables by age.

	<1 Year of Age *n* = 58	>1 Year of Age *n* = 80	*p*-Value
Mechanical ventilation (days)	13 (2.0–31.7)	0.5 (0.5–2.0)	<0.0001
PICU LOS (days)	20.3 (9.0–48.2)	3.9 (2.0–7.8)	<0.0001
Hospital LOS (days)	62 (26.0–133.0)	12.6 (7.3–28.0)	<0.0001
Stunting (HFA < −2 z-score), *n* (%)	13 (16.3)	17 (29.3)	0.0936
Underweight (WFA < −2 z-score), *n* (%)	6 (10.3)	8 (10.0)	>0.9999
Acute malnutrition (WFL < −2 z-score), *n* (%)	2 (3.5)	0 (0)	0.1749

Continuous variables are represented as medians with interquartile ranges (25th–75th) and compared by Mann–Whitney test. PICU: pediatric intensive care unit; LOS: length of stay; HFA: height-for-age, WFA: weight-for-age, WFL: weight-for-length. Categorical variables are expressed as numbers and percentages and compared using Fisher’s Exact test.

## Data Availability

The original contributions presented in the study are included in the article; further inquiries can be directed to the corresponding author due to privacy reasons.

## Abbreviations

The following abbreviations are used in this manuscript:

ASPEN	American Society of Parenteral and Enteral Nutrition
BMI/A	Body Mass Index-For-Age
CDC	Centers for Disease Control and Prevention
EMR	Electronic Medical Records
G/Kg/Day	Grams Per Kilogram Per Day
HFA	Height/Length-For-Age
Kcal/Kg/Day	Kilocalories Per Kilogram Per Day
LOS	Length of Stay
MV	Mechanical Ventilation
PELD	Pediatric End-Stage Liver Disease
SCCM	Society Of Critical Care Medicine
SD	Standard Deviation
WFA	Weight-For-Age
WHO	World Health Organization
